# Regeneration of Pancreatic β-Cells for Diabetes Therapeutics by Natural DYRK1A Inhibitors

**DOI:** 10.3390/metabo13010051

**Published:** 2022-12-29

**Authors:** Yichuan Guo, Lingqiao Li, Yuanfa Yao, Hanbing Li

**Affiliations:** 1 Institute of Pharmacology, College of Pharmaceutical Sciences, Zhejiang University of Technology, Hangzhou 310014, China; 2 Zhejiang Starry Pharmaceutical Co., Ltd., Taizhou 317306, China

**Keywords:** diabetes, DYRK1A inhibitor, β-cell, proliferation, natural products

## Abstract

The pathogenesis of diabetes mellitus is characterized by insulin resistance and islet β-cell dysfunction. Up to now, the focus of diabetes treatment has been to control blood glucose to prevent diabetic complications. There is an urgent need to develop a therapeutic approach to restore the mass and function of β-cells. Although exogenous islet cell transplantation has been used to help patients control blood glucose, it is costly and has very narrow application scenario. So far, small molecules have been reported to stimulate β-cell proliferation and expand β-cell mass, increasing insulin secretion. Dual-specificity tyrosine-regulated kinase 1A (DYRK1A) inhibitors can induce human β-cell proliferation in vitro and in vivo, and show great potential in the field of diabetes therapeutics. From this perspective, we elaborated on the mechanism by which DYRK1A inhibitors regulate the proliferation of pancreatic β-cells, and summarized several effective natural DYRK1A inhibitors, hoping to provide clues for subsequent structural optimization and drug development in the future.

## 1. Introduction

The new version of the International Diabetes Federation (IDF) Diabetes Atlas provides data on the prevalence of diabetes, its associated morbidity, mortality, and healthcare costs in various nations and areas around the world. In 2021, an estimated 537 million people worldwide between the ages of 20 and 79 had diabetes, accounting for 10.5% of the total population in that age group. By 2030, this figure is projected to increase to 643 million. Among the 537 million individuals with diabetes, about 240 million remain undiagnosed, and 75% live in low- and middle-income nations, which indicates that too many people do not receive quality care. Diabetes can be classified as type 1 diabetes mellitus (T1DM), type 2 diabetes mellitus (T2DM), gestational diabetes, or other types of diabetes depending on its characteristics. The etiology of T1DM, also known as autoimmune diabetes, is not fully understood, but T-cell mediated β-cell destruction is one of the recognized pathogeneses [1]. T2DM is the most common type of diabetes, accounting for more than 90% of the global total. It is characterized by reduced sensitivity of target organs to insulin, resulting in a relative lack of insulin secretion. Long-term insulin resistance leads to disorders of glucolipid metabolism and damage to islet β-cells, and ultimately absolutely insufficient insulin secretion [2]. Thus, both T1DM and T2DM involve a reduction in islet β-cell mass, and human islet β cells do not replicate after birth. Hence, there is an urgent need for antidiabetic therapy that can increase the mass of adult β-cells or induce their regeneration besides existing therapeutics.

At present, the research strategy of restoring β-cell mass in diabetic patients includes pancreas transplantation, pancreatic islet transplantation [3,4], stem cell transplantation [5], and using small molecules to promote the proliferation of islet β-cells. Among them, the application of pancreas and islet transplantation is very limited and requires careful patient evaluation before transplantation. Pancreas transplantation is more suitable for patients with severe diabetic nephropathy and is usually carried out simultaneously with kidney transplantation. Islet transplants are usually performed in patients whose blood glucose remains abnormal even after insulin or other drug interventions. Furthermore, both pancreas and islet transplantation face many other limitations, such as lack of donors, high cost, and lifelong immunosuppressant application, which prevent them from being widely used. Transplantation of stem-cell-derived pancreatic endoderm cells can reduce patients’ dependence on insulin treatment. However, stem cell transplantation is still experimental and faces the same problems as pancreas and islet transplantation. In addition to these three types of transplants mentioned above, using small molecules to promote islet β-cell proliferation to increase insulin secretion, thereby improving patient blood glucose control, has been documented, and shows greater potential. Under normal conditions, human β-cells have a very low proliferation rate, thus it is necessary to deepen the understanding of signaling pathways that affect the proliferation rate of β-cells. Targets that can affect the cell cycle of islet β cells include glucagon-like peptide-1 (GLP-1) receptor [6], dual-specificity tyrosine-regulated kinase-1A (DYRK1A) [7], transforming growth factor-β receptor (TGF-βR) [8], glycogen synthase kinase-3β (GSK3β) [9], the phosphatidylinositol 3 kinase (PI3K)-serine-threonine protein kinase B (Akt)-mammalian target of rapamycin (mTOR) signaling pathway [10], serotonin receptor 2B (HTR2B) [11], and diacylglycerol kinase δ (DGKδ) [12]. So far, only GLP-1R agonists are on the market, but their effect on the proliferation of human β cells is very low.

The coding gene of DYRK1A, located in chromosome 21 Down Syndrome Critical Region (DSCR), belongs to the CMGC family of evolutionally highly conserved protein kinases. The protein consists of 763 amino acids, including six domains: two nuclear localization signal (NLS) domains, a kinase functional domain, a carbon-terminal PEST region, a multi-histidine bundle and a serine/threonine enrichment region. Tyrosines at positions 319 and 321 are the key sites for its complete catalysis, which can phosphorylate not only on serine and threonine, but also on tyrosine. In mammals, the DYRK family includes five subtypes: DYRK1A, DYRK1B, DYRK2, DYRK3 and DYRK4. Among them, DYRK1A is the most expressed protein kinase, and plays an important role in physiological and pathological processes such as neural development [13], cell proliferation and differentiation [14], and tumorigenesis [15]. The inhibition of DYRK1A could be a promising target to restore islet β-cell dysfunction in diabetics. Wang et al. first discovered that harmine can promote the proliferation of human islet β-cells through high-throughput small-molecule screens (HTS) in 2015, and identified its target DYRK1A [16]. Since then, more and more small molecular DYRK1A inhibitors have been found to increase the mass of islet β-cells, thereby alleviating diabetes. In this paper, we will discuss the regulative roles of DYRK1A in islet β-cells and the mechanism of its inhibitors in improving diabetes, and introduce some natural DYRK1A inhibitors that are being studied.

## 2. Factors Causing Islet β-Cell Damage

As previously mentioned, both T1DM and T2DM involve the loss of islet β cell mass, but the reasons are distinct. T1DM is mostly caused by islet β-cell autoimmunity influenced by genetic and environmental factors, resulting in absolute lack of β-cell mass and insulin deficiency and often occurs in childhood or adolescence [17]. The etiology of T1DM is not fully understood, but the pathogenesis of T1DM is thought to involve T cell-mediated β-cell destruction. Autoantibodies against insulin, GAD65, protein tyrosine phosphatase-like molecules IA-2, IA-2β and ZNT8 are produced by B cells. CD8^+^ and CD4^+^ T cells are activated to initiate an autoimmune response to islet β-cells, and the dysregulation of glucose metabolism caused by the loss of islet β-cells can further damage the existing β-cells through endoplasmic reticulum stress and oxidative stress [18].

**Figure 1:**
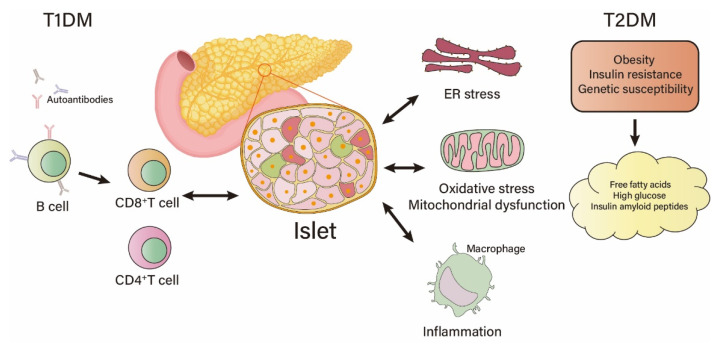
Islet β-cell dysfunction during the pathogenesis of type 1 diabetes mellitus (T1DM) and type 2 diabetes mellitus (T2DM).

Although T2DM does not directly cause islet β-cell damage at the initial stage of onset, the mass of islet β-cells decreases with the duration of clinical diabetes. In subjects with diabetes <5 years and >15 years, the mass of β cells is 24% and 54% lower than that in a control group, respectively [19]. Due to genetic differences, some individuals are more sensitive to environmental factors. Genome-wide association studies (GWAS) have shown that many genes are closely related to individual genetic susceptibility to diabetes, such as *ABO*, *IGF2BP2*, *MTNR1B*, *TCF7L2*, *HNF1A*, *HNF1B*, *ADCY5*, *SLC30A8*, *CCND2* and *PAM*, which make an individual more vulnerable to islet dysfunction [20]. During the pathogenesis of T2DM, imbalance in glucolipid metabolism occurs [21], which plays an important role in the progressive β-cell dysfunction [22]. Impaired insulin sensitivity in peripheral tissues and long-term unhealthy lifestyle, such as lack of exercise and consuming a high-fat and high-sugar diet, can lead to elevated free fatty acids (FFA) and blood glucose, also known as glucolipotoxicity, which affects islet β-cell function in a variety of ways. Accumulation of unfolded proteins induced by FFA, high glucose, and islet amyloid polypeptide (IAPP) in the endoplasmic reticulum (ER) of pancreatic β-cells recruit ER chaperone BIP. BIP dissociates from PREK, IRE1 and ATF6 receptors, thereby affecting the expression of related genes and impairing mitochondrial function [23] (Figure 1). FFA induces hydrogen peroxide production in β-cells, influencing mitochondrial β-oxidation [24]. Additionally, FFA and high glucose act on NADPH oxidase to produce superoxide, and both hydrogen peroxide and superoxide further cause oxidative stress in β-cells [25]. Saturated FFAs, such as palmitate, induce the production of cytokines IL-1B, IL-6, IL-8 and chemokines CCL2 and CXCL1 in β-cells, which further lead to oxidative stress and endoplasmic reticulum stress. Palmitate also triggers the production of chemokines through Toll-like receptor 4 (TLR4), which recruits M1-type pro-inflammatory macrophages and monocytes to the islets [26]. Palmitate and high glucose could synergistically trigger the secretion of S100 calcium-binding protein A8 (S100A8), a damage-related model molecule constitutively expressed in neutrophils and activated macrophages. Subsequent production of cytokines by macrophages induces β-cell apoptosis (Figure 1). Glucose can increase the expression of c-Myc in islet β-cells. C-Myc, a transcription factor related to cell growth, proliferation, apoptosis, organogenesis and metabolism, can play different roles depending on its level in islet β-cells [27]. Under normal physiological conditions, c-Myc maintains a very low level, while supraphysiological rise of c-Myc induced by persistent hyperglycemia or other factors in β-cells suppresses the insulin gene transcription by inhibiting NeuroD-mediated transcriptional activation [28] and induces β-cell dedifferentiation or insulinoma formation [29]. To sum up, insulin resistance and β-cell failure are the core pathophysiologic features during the development of T2DM.

## 3. Role of DYRK1A in the Proliferation and Function of Islet β-Cells

DYRK1A is a potential therapeutic target for many diseases including Down Syndrome (DS) [30,31], Alzheimer’s disease (AD) [32,33] and Parkinson’s disease (PD) [34]. DYRK1A also plays an important role in the survival and proliferation of many tumor cells [35,36,37]. In addition, DYRK1A is involved in the replication of a variety of viruses [15,38,39], suggesting that the inhibitor has an antiviral effect.

**Figure 2:**
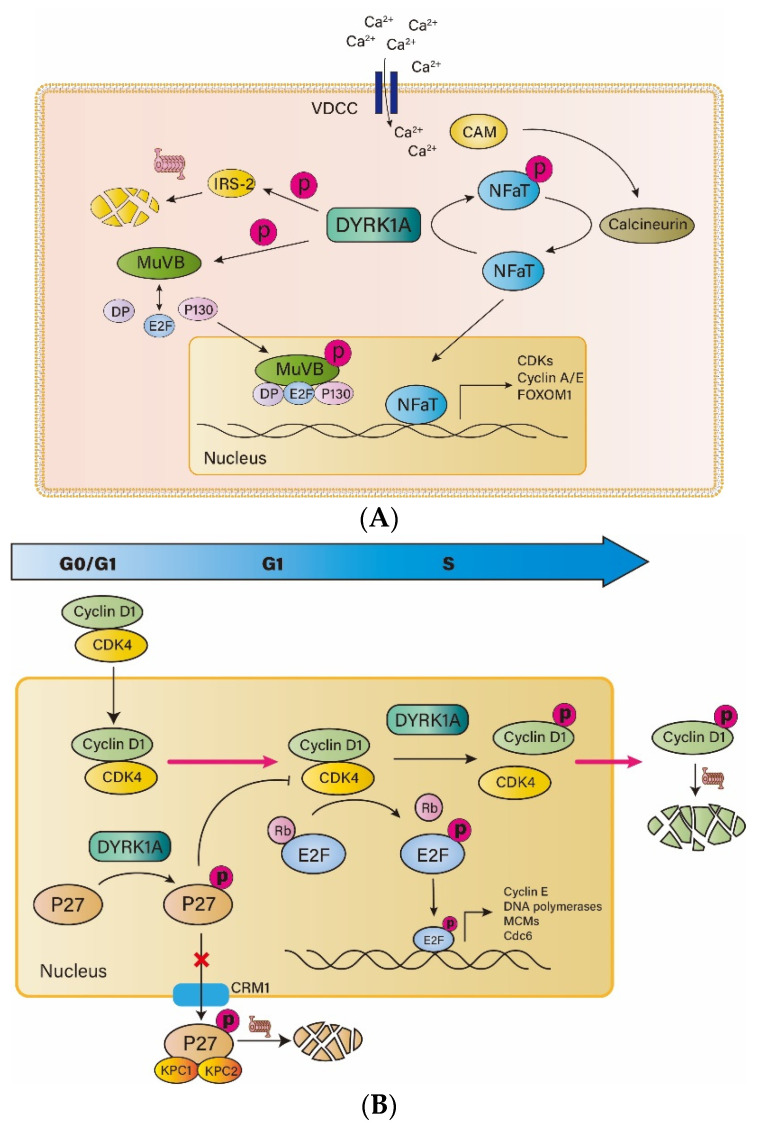
DYRK1A regulates the cell cycle. (**A**) DYRK1A inhibits islet β-cell proliferation by inhibiting NFAT entry into the nucleus, promoting DREAM formation and phosphorylation of IRS-2 for degradation. (**B**) DYRK1A phosphorylates P27 at the G0/G1 phase to stabilize it and prevent its nucleation. In addition, DYRK1A phosphorylates Cyclin D1 during the S phase, promoting its nucleation and degradation. VDCC, voltage-dependent calcium channels; IRS-2, insulin receptor substrate-2; DYRK1A, dual-specificity tyrosine-regulated kinase 1A; NFAT, nuclear factor of activated T cell; CAM, calmodulin; CDK4, cyclin dependent kinase 4; E2F, adenoviral early region 2 binding factor; Rb, retinoblastoma; CRM1, chromosome region maintenance 1; KPC1/2, KIP1 ubiquitination-promoting complex1/2.

In the field of diabetes, many breakthroughs have been made in the discovery of DYRK1A inhibitors, including harmine, 5-IT and GNF2877, which can significantly promote the proliferation of rodent and human β-cells. The potential pathways by which DYRK1A affects the proliferation and function of islet β-cells are summarized below (Figure 2A,B).

### 3.1. DYRK1A and NFAT

The phosphorylation of nuclear factor of activated T cell (NFAT) is one of the most reported ways that DYRK1A inhibits the proliferation of β-cells. Transcription factors of NFAT family promote cell cycle entry by increased transcription of cell cycle activating genes (such as *CCNE*, *CCNA* and *CDK1*) and decreased transcription of cell cycle suppressors (such as *CDKN1C*, *CDKN2A* and *CDKN2B*). However, NFAT binds to 14-3-3 proteins in the form of phosphorylation before activation and thus is immobilized in the cytoplasm. Its entry into the nucleus requires the activation of calcium-calmodulin (CAM)-calcineurin (CnA and CnB)-NFAT pathway. Voltage-dependent calcium channels (VDCC) open in response to appropriate stimulation, such as glucose, sulfonylureas, and the GLP-1 family, allowing extracellular calcium ions to enter cells along the concentration gradient. The increase of calcium ion concentration directly activates CAM, which in turn activates CnA and CnB. Activated CnA and CnB form a phosphatase complex and dephosphorylate NFAT. Dephosphorylated NFAT is separated from the 14-3-3 protein and is transferred to the nucleus, where it binds to DNA and regulates the expression of related genes. DYRK1A phosphorylates NFAT and prevents it from entering the nucleus, thereby blocking its regulatory role in promoting entry into the cell cycle [40].

### 3.2. DYRK1A and IRS2

Insulin receptor substrate-2 (IRS2) is an important intermediate protein in the insulin signaling pathway, and plays an indispensable role in the survival and function of β cells [41,42]. IRS2 can be activated by peptide ligands such as insulin and IGF-1, and then affects the Ras-Raf-MAPK pathway, which phosphorylates ERK1/2 and upregulates expression of MAFA, PDX1 and other proteins closely related to β-cell function. IRS2 also affects the PI3K-Akt pathway [43], which in turn affects the expression of FOXO1, P27, P21 and other genes, which are essential for the survival and proliferation of β-cells. DYRK1A interacts directly with IRS2 to promote phosphorylation of IRS2, leading to degradation of the IRS2 proteasome, resulting in β-cell dysfunction and apoptosis [44].

### 3.3. DYRK1A and DREAM

The DREAM complex is composed of dimerization partner (DP), retinoblastoma (RB), E2F and five stable core complexes of MuvB-like proteins (LIN9, LIN37, LIN52, LIN54 and RBBP4) [45]; its formation inhibits the function of E2F in regulating gene transcription. E2F plays an important role in the survival and replication of β cells, and E2F1 (−/−) mice exhibit obvious pancreatic shrinkage and impaired islet function [46]. DYRK1A specifically phosphorylates the serine residue 28 of LIN52, a step required for DREAM complex formation [47]. Inhibition of the DYRK1A active site of LIN52 may disrupt the formation of the DREAM complex, release E2F and promote cell-cycle entry. These reveal the important role of DYRK1A in regulating the activity of the DREAM complex and entering the quiescent state.

### 3.4. DYRK1A and P27

The cell cycle regulator P27, a kind of CDK inhibitor (CKI), plays a crucial role in the formation of the mass of β-cells in an individual before birth. The proliferation of β-cells requires a balance between cyclin-CDK and CKI. P27 inhibits the cyclin-CDK complex and keeps cells in the quiescent phase. Previous studies suggest that KIS phosphorylation of P27 at ser10 makes P27 bind to its outlet protein CRM1 on the nuclear membrane, thus transferring out of the nucleus, and then is hydrolyzed by proteasome in the cytoplasm, making P27 unable to regulate the cell cycle [48]. However, recent studies have shown that DYRK1A phosphorylates P27 at Ser10, stabilizes P27 and increases its concentration in the nucleus [49,50]. DYRKs phosphorylation of P27 occurs at G0 phase, while KIS phosphorylation of P27 occurs at G1 phase. Mitogen can downregulate the expression of DYRKs and upregulate the expression of KIS, so that the two types of phosphorylation occur at different times. The phosphorylation of the same site of P27 by the two enzymes has opposite effects at different time points. Therefore, inhibition of DYRK1A can downregulate the concentration of P27 in the G0/G1 phase in the nucleus, thereby preventing the inhibition of P27 on the cell cycle.

### 3.5. DYRK1A and Cyclin D

Cyclin D1 is an important factor regulating the proliferation of β-cells [51]. After receiving an extracellular mitosis signal, cyclin D1 is activated by RAS, Wnt and NF-κB signaling pathways. Then, cyclin D1 will bind to CDK4 and enter the nucleus in response to CIP/KIP. Upon entry into the nucleus, RB is phosphorylated by the cyclin D1-CDK4 complex, leading to the release of E2F, which regulates transcription of cell-cycle-related genes such as DNA polymerases, small chromosome maintenance complex components (MCMs), CDC6, and cyclin E, and promotes cell cycle progression [52]. In addition, the cyclin D1-CDK4 complex phosphorylates SMAD3 and FOXM1 to promote cell cycle progression. DYRK1A phosphorylates cyclin D1 at Thr286 in the S phase of the cells, promoting cyclin D1 to leave the nucleus and be degraded by the proteasome [49].

## 4. DYRK1A Inhibitors from Natural Products

So far, no drug targeting human islet β-cell proliferation has been put on the market. However, many compounds that could induce regeneration of human β-cells have been investigated, including osteoprotegerin [53], denosumab [54], parathyroid hormone related protein [55,56], Serpin B1 [57], the peptide TLQP21 [58] and γ-aminobutyric acid [59]. In general, the β-cell proliferation rate induced by these molecules is 0.5–1%. DYRK1A inhibitors have been shown to induce human β-cell proliferation in the 1–3% range, including harmine [16,60], INDY [61], GNF4877 [62], 5-IOdotubericidin (5-IT) [63], CC-401 [50], and others [64], indicating that DYRK1A inhibitors have great potential to improve diabetes due to their ability to promote β-cell proliferation. In view of the diversity of chemical structures, phytochemicals provide an alternative source for potential lead compounds inhibiting DYRK1A (Table 1).

### 4.1. Harmine and Its Derivatives

Harmine is derived from the seeds of the medicinal plant *Peganum harmala* L. which grows in arid regions, such as the Middle East and some provinces of China, and has long been widely used in folk medicine. Harmine has a broad spectrum of anti-inflammatory [74] and anti-tumor activities [75], and has also shown beneficial effects upon cognitive diseases such as Alzheimer’s disease [76,77] and Down Syndrome [78]. In 2015, researchers first discovered that harmine can promote mitosis of human islet β-cells by labeling of Ki67, BrdU, and insulin markers [16]. Harmine increased rat islet cell proliferation by about 8% and human islet β-cell proliferation by about 1% to 3%, indicating potential application for diabetic therapeutics, given that adult human β-cells are mostly quiescent and exhibit extremely low levels of proliferation capacity. In terms of mechanisms, harmine promoted the proliferation of β-cells by upregulating the gene expression related to the cell cycle via inhibiting the phosphorylation of NFAT by DYRK1A.

Furthermore, the introduction of hydroxymethyl at 1-position improved the selectivity of harmine for DYRK1A, and significantly decreased their affinity for DYRK1B and CLK1 without significantly affecting their ability to promote β-cell proliferation [65]. Introduction of carboxamide at N9 position of harmine can significantly promote islet β-cell proliferation, and enhance its kinase selectivity, reduce the interaction with 5-hydroxytryptamine and tryptamine, and ameliorate the influence of harmine on the central nervous system.

The combination of harmine with other agents can significantly improve its proliferative effect on islet β-cells or enhance its islet β-cell selectivity. When combined with TGFβ superfamily inhibitors, harmine increased the number of ki-67 labeled β-cells from 1–3% to 5–8%. Endogenous ligands such as TGFβ and BMPs activate TGFβ receptors, trigger phosphorylation of Smad2/3 and Smad1/5/9 bound to Smad4, and form the trithorax complex with KDM6A and MEN1 in the nucleus, thus upregulating the expression of CDKIs protein and inhibiting the cell cycle. Co-inhibition of the DYRK1A and TGFβ signaling pathway showed a significant synergistic effect in promoting β-cell proliferation by regulating the gene expression related to cell cycle [79]. Analogously, the combination of GLP-1 receptor agonist (GLP-1RA) and harmine can significantly increase the proliferation rate of β-cells, and this synergistic effect mainly occurs in cells with high expression of GLP-1R, which makes the selectivity of harmine relatively enhanced. On the mechanism, the synergistic effect of GLP-1RA and harmine was due to the increase of intracellular cAMP concentration upon GLP-1RA. Inhibition of PKA or EPAC2, downstream of cAMP, would not affect the proliferative effect of harmine itself, however, it abolishes the synergistic effect of GLP-1RA and harmine [80]. Given the limited selectivity of harmine, the combination of harmine with other drugs might be a potential therapeutic alternative against diabetes in the future.

### 4.2. Epigallocatechin-3-Gallate (EGCG)

As one of the most popular beverages in the world, tea contains many active pharmacological molecules. Epicatechin-3-gallate (EGCG) is a major polyphenolic compound in green tea and one of the most widely studied catechins in green tea. A plethora of in vivo and in vitro evidence shows that catechins have good effects on a variety of pathological diseases, such as cancer, diabetes and cardiovascular disease [81]. Against diabetes, EGCG increases the number and the size of islets and improves glucose tolerance and increases glucose-stimulated insulin secretion [82]. Moreover, EGCG could bind to α-amylase and α-glucosidase in the intestine, thereby inhibiting the hydrolysis of starch [67], and increase glucose uptake in C2C12 myotubes by activating AMPK pathway [83]. EGCG also could inhibit adipogenesis, evoke white adipocyte beiging and relieve the blockage of insulin signaling pathway in 3T3-L1 cells induced by TNF-α [68]. In addition, it was reported that EGCG could inhibit DYRK1A with an IC_50_ of 0.33 mM, while it exhibited low affinity for other kinases in the CMGC family, such as MAPK and GSK3β [84], indicating its distinct potential against diabetes. To sum up, EGCG can increase the mass of islet β cells and improve diabetes, but no study has linked its inhibitory effect on DYRK1A to diabetes.

### 4.3. Desmethylbellidifolin (DMB)

Desmethylbellidifolin (DMB) is a natural flavonoid extracted from *Gentianella acuta* [85], and plays an antidiarrheal role in traditional Mongolian medicine. DMB may inhibit ulcerative colitis induced by TNBS in Sprague Dawley rats and Kunming mice, reducing the inflammatory response and relieving colonic muscle spasms [86]. DMB was found to be a potent DYRK1A inhibitor with IC_50_ of 370 nM by molecular docking. DMB significantly enhanced the proliferation of INS-1 cells in the range of 2–75 μM with or without STZ treatment, and the proliferation effect was proportional to the dose. In terms of the mechanism, NFATc1 was transferred from the cytoplasm to nucleus after DMB treatment, and the expressions of cyclin D1, cyclin D2 and cyclin D3 were dose-dependently upregulated after DMB treatment, while the expressions of *p15^INK4^*, *p16^INK4^* and *p57^CIP2^* decreased. In addition, DMB induced the phosphorylation of Smad3 and decreased the expression of FOXO1 in INS-1 cells, suggesting that the effect of DMB on the proliferation of islet β-cells may be related to the inhibition of TGFβ pathway [69].

### 4.4. 2-Aminoimidazolone Alkaloids

2-Aminoimidazolone alkaloids, a class of natural products extracted from sponges *Leucetta* and *Clatrina*, are effective inhibitors of DYRKs and CDC2-like kinases (CLKs), and have great potential as lead compounds for the remission of related diseases [87]. Polyandrocarpamines A/B and Leucettine L41 were identified as the most potent DYRK1A inhibitors by testing a series of 2-aminidazolidone alkaloids. The IC_50_ of Leucettine L41 was 32 nM for DYRK1A, and Polyandrocarpamines A/B were 270 nM and 470 nM. Polyandrocarpamines A significantly downregulated the phosphorylation of cyclin D1 at Thr286 in sh-SY5Y cellz, preventing the translocation of cyclin D1 from the nucleus to the cytoplasm, where it was degraded [70]. Phosphorylation of Thr286 of cyclin D1 is also an important step in influencing the islet β-cell cycle, so 2-aminoimidazolone alkaloids are worthy of further exploration in the field of diabetes.

### 4.5. Aristolactam BIII

Aristolochyllactam is an alkaloid containing phenanthrene, which is mainly found in herbaceous plants, such as aristolochylaceae, annonaceae, moniaceae, pinellidae and piperaceae. Aristolocholate BIII mainly exists in the stem of *Fissistigma oldhamii* (FO), which is a medicinal plant with functions of removing dampness, promoting blood circulation and relieving pain [88], The aristolochic acid BIII showed moderate antitumor activity [89] and strong inhibitory activity against platelet aggregation induced by thrombin, collagen, and platelet activators in rabbits [90]. Interestingly, aristolactam BIII also effectively inhibited DYRK1A activity (IC_50_ = 9.67 nM) and Tau phosphorylation in mammalian cells mediated by DYRK1A. It is a specific DYRK1A inhibitor, with low inhibition rates for other members of the CMGC family, such as DYRK4 (IC_50_ = 1184 nM), CLK1 (IC_50_ = 50.28 nM), and GSK-3β (IC_50_ = 240.3 nM). At the same time, aristolochic amide BIII significantly increased cyclin D1 content in primary fibroblasts of DYRK1A TG mice and increased BrdU labeling by 2–3 times. Experiments further demonstrated that aristolactam BIII could ameliorate cognitive impairment in DYRK1A overexpression mice in vivo [71]. At present, aristolactam BIII has not been studied in the field of diabetes, but its structure–activity relationship can provide an idea for us to develop DYRK1A inhibitors targeting islet β-cells.

### 4.6. 4-Cresol

4-Cresol is a product of protein metabolism in mammals, produced by intestinal flora metabolism, and is also widely found in food (smoked food, tomatoes, asparagus, dairy), beverages (coffee, tea, wine), cigarette smoke, wood burning, and in surface water and groundwater. It can be absorbed through the digestive tract, respiratory or skin contact, affecting the occurrence and development of a variety of diseases. Free 4-cresol caused by chronic kidney disease has a significant correlation with the occurrence probability of cardiovascular diseases [91]. Through metabolome profiling, serum 4-cresol is negatively correlated with many indicators of diabetes. Chronic non-toxic doses of 4-cresol can reduce obesity, glucose intolerance and liver triglycerides, and enhance insulin secretion. In terms of mechanisms, 4-cresol stimulates proliferation of pancreatic β-cells, by downregulation of DYRK1A, which mediates its biological effects [72].

### 4.7. Licocoumarone

*G. uralensis* Fisch, a traditional Chinese medicine, has been used for thousands of years to treat coughs, bronchitis and peptic ulcers. Many studies of *G. uralensis* Fisch extract suggest it can play a greater role in modern medicine, including type 2 diabetes [92,93]. Licocoumarone, as a component in *G. uralensis* Fisch, was found to have a strong inhibitory effect upon DYRK1A with IC_50_ of 12.56 μM. Licocoumarone reduced the c-Met protein level in BxPC3 cells by inhibiting DYRK1A, thereby inhibiting cell proliferation and migration, and ultimately inhibiting the survival of cancer cells [73]. Whether its DYRK1A inhibitory effect contributes to anti-diabetic action needs to be elucidated.

## 5. Conclusions and Perspectives

Oral hypoglycemic agents currently on the market include metformin, sulfonylureas, meglitinides thiazolidinediones, α-glucosidase inhibitors, dipeptidyl peptidase 4 (DPP4) inhibitors, sodium-glucose cotransporter-2 (SGLT2) inhibitors and GLP-1RA. They can reduce hyperglycemia by promoting insulin secretion, inhibiting glucose absorption in the digestive tract, promoting insulin sensitivity and glucose uptake in peripheral tissues, and preventing kidneys from reabsorbing glucose. However, no drug can specifically reverse the decrease of islet β-cell mass, although several drugs and hormones such as GLP-1RA, IGF-1, and HGF have been inspected for their ability to stimulate β-cell proliferation [94]. This unmet clinical need warrants the discovery of new drug therapeutic approaches. DYRK1A affects the cell cycle of β-cells through a variety of pathways, including phosphorylation of transcriptional factors such as NFAT, P27, DREAM complex and cyclin D1, thus restoring the β-cell mass and function. Therefore, as an effective β-cell proliferation promotor, DYRK1A inhibitors are of great research value. Phytochemicals from natural products possessing diverse chemical structures and DYRK1A inhibitory properties have been reported, providing potential candidates for improving β-cell dysfunction and promoting β-cell biogenesis.

Although great progress has been made in the application of DYRK1A inhibitors to improve β-cell mass and function, there are still many problems that need to be addressed if we want DYRK1A inhibitors to truly benefit the majority of diabetic patients. First, the methods to evaluate islet β-cell proliferation rate are mainly calculated by Ki67, EDU, and BrdU staining, which can mark cells in replication at a certain time or within a short period of time and might not necessarily reflect the real change of islet β-cell mass. We need to explore new methods to evaluate the actual change of β-cell mass and whether they can improve blood glucose control. Second, although DYRK1A inhibitors have exhibited strong anti-diabetic potential in vivo and in vitro, these compounds face severe challenges. DYRK1A is widely distributed in various tissues throughout the body, and can play a critical role in a variety of biological processes including cell survival and proliferation, such as B-cell survival [95,96], HaCaT cell proliferation [97], the development and aging of the central nervous system (CNS) [98], and lipid metabolism [99] Therefore, we must address the problem to enhance the selectivity of compounds for islet β-cells. Taking EGCG as an example, this can easily pass the blood–brain barrier and exert pharmacological effects on the central nervous system, which is an important obstacle to their further study in the field of diabetic therapeutics. By structural modification, we can add a β-cell targeting ligand, such as ectonucleoside triphosphate diphosphohydrolase-3 (NTPDase3) antibody [100], to the compound to enhance the targeting of the compound and reduce the side effect of DYRK1A inhibitor on non-islet β-cells. Meanwhile, the kinase profiles of current DYRK1A inhibitors indicate a lack of selectivity for CMGC family kinases in general. In particular, DYRKs and CLKs, two highly conserved and related kinase families, are usually sensitive to the same present inhibitors. Therefore, these natural DYRK1A inhibitors require more complex genetic, chemical biology, and pharmacology studies in the future, to develop inhibitors that are more specific to DYRK1A and have fewer off-target effects. Third, the proliferation rate of human β-cells is very low, which is a serious challenge before us. This is distinct from rodents and might lead to the possibility that effective compounds obtained in rodents may not achieve the same efficacy in humans. Breakthroughs in the understanding of the pathways regulating human pancreatic β-cell proliferation and better animal models mimicking the pathogenesis of diabetes would facilitate the finding of better DYRK1A inhibitors, leading to smooth translation into clinical investigation and final drug approval. Fourth, cell cycle regulators can easily lead to insulinoma and undesired proliferation or oncogenic transformation. Specifically, DYRK1A under physiological conditions is an important factor in maintaining a stable low level of c-Myc in islet β-cells. Supraphysiologically increased expression of c-Myc may lead to β-cell death, dedifferentiation, or the formation of insulinomas [27,101]. Therefore, while exploring the promotion of β-cell proliferation by various natural DYRK1A inhibitors, more attention should be paid to whether it is likely to cause the above problems in clinical applications. Whether natural DYRK1A inhibitors are safe and reliable tissue-expanding agents remains to be determined by ongoing and future preclinical and clinical studies. Finally, DYRK1A inhibitors increase insulin secretion by promoting islet β-cell proliferation without affecting insulin resistance and autoimmunity, and, given that the pathogenesis of diabetes is a complex and multifactorial contributing process, the combination of DYRK1A inhibitors with other anti-diabetic drugs would be an alternative to maximize their role in diabetes therapeutics.

## Figures and Tables

**Table 1:** Regeneration of pancreatic β-cells for diabetes therapeutics by natural DYRK1A inhibitors.

Compound	Source	IC_50_ for DYRK1A	Model		Effects	Ref.
Harmine	*Peganum harmala* L.	28 nM	In vitro	RIN-m5f INS-1 Human β-cells	Increasing 8% Ki67 labeling for rat β-cells. Increasing 1% Ki67 labeling for human β-cell at 10 μM.	[16]
			In vivo	Human islet transplanted in STZ-diabetic NOD-SCID mice	Increasing 3-fold Ki67 labeling for human β-cells.	
Harmine Analogue 2-2		54.8 nM	In vitro	Human islet	Increasing 2.5% Ki67 labeling for human β-cells at 30 μM.	[65]
Harmine Analogue 2-2c		25 nM	In vitro	Human islet	Increasing 1.5% Ki67 labeling for human β-cells at 3 μM.	[66]
			In vivo	Human islet transplanted in NOD-SCID mice	Increasing 1.75% Ki67 labeling for human β-cells.	
Epicatechin-3-gallate	Green tea	330 nM	In vitro	HepG2 and 3T3-L1 cells	Suppressing oxidative stress and regulating mitochondrial function.	[67,68]
			In vivo	T2DM mice induced by high-fat diet and streptozotocin induced diabetes mice	Repressing gluconeogenesis and lipogenesis in the liver.	
Desmethylbellidifolin (DMB)	*Gentianella acuta*	370 nM	In vitro	INS-1 cells	Increasing 30% EDU labeling for rat β-cells.	[69]
			In vivo	db/db mice	Increasing 6% ki67 labeling for rat β-cells.	
Polyandrocarpamines A Polyandrocarpamines B Leucettine L41	Marine Calcareous sponges *Leucetta* and *Clathrina*	270 nM 470 nM 32 nM	In vitro	SH-SY5Y neuroblastoma cells	Increasing Thr286-cyclin D1 phosphorylation.	[70]
Aristolactam BIII	*Fissistigma oldhamii*	9.67 nM	In vitro	Primary fibroblast cells of DYRK1A TG mice	Decreasing cyclin D1 at 100 nM. 2–3 fold increase in BrdU labeling at 100 nM.	[71]
			In vivo	DYRK1A TG mice	Lowering Tau phosphorylation in the hippocampus and frontal cortex. Improving the locomotive and exploratory behavior of DYRK1A-overexpressing mice in the open field test.	
4-Cresol	Metabolite produced by intestinal bacteria	ND	In vitro	Primary islet cells of c57BL/6 mice	Increasing 2.38-fold ki67 labeling at 10 nM.	[72]
			In vivo	Mice fed high-fat diet	Increasing glucose-stimulated secretion of insulin, Reducing liver triglycerides.	
Licocoumarone	*Glycyrrhiza uralensis* Fisch	12.56 μM	In vitro	Human pancreatic adenocarcinoma cell line (BxPC-3)	Suppressing proliferation and inducing cell apoptosis.	[73]

## References

[1] Katsarou A., Gudbjornsdottir S., Rawshani A., Dabelea D., Bonifacio E., Anderson B.J., Jacobsen L.M., Schatz D.A., Lernmark A. (2017). Type 1 diabetes mellitus. Nat. Rev. Dis. Primers.

[2] Inaishi J., Saisho Y. (2020). Beta-Cell Mass in Obesity and Type 2 Diabetes, and Its Relation to Pancreas Fat: A Mini-Review. Nutrients.

[3] Marfil-Garza B.A., Shapiro A.M.J., Kin T. (2021). Clinical islet transplantation: Current progress and new frontiers. J. Hepatobiliary Pancreat. Sci..

[4] Wu S., Wang L., Fang Y., Huang H., You X., Wu J. (2021). Advances in Encapsulation and Delivery Strategies for Islet Transplantation. Adv. Healthc. Mater..

[5] Ramzy A., Thompson D.M., Ward-Hartstonge K.A., Ivison S., Cook L., Garcia R.V., Loyal J., Kim P.T.W., Warnock G.L., Levings M.K. (2021). Implanted pluripotent stem-cell-derived pancreatic endoderm cells secrete glucose-responsive C-peptide in patients with type 1 diabetes. Cell Stem Cell.

[6] Buteau J. (2008). GLP-1 receptor signaling: Effects on pancreatic beta-cell proliferation and survival. Diabetes Metab..

[7] Kumar K., Suebsuwong C., Wang P., Garcia-Ocana A., Stewart A.F., DeVita R.J. (2021). DYRK1A Inhibitors as Potential Therapeutics for beta-Cell Regeneration for Diabetes. J. Med. Chem..

[8] Lee J.H., Mellado-Gil J.M., Bahn Y.J., Pathy S.M., Zhang Y.E., Rane S.G. (2020). Protection from beta-cell apoptosis by inhibition of TGF-beta/Smad3 signaling. Cell Death Dis..

[9] Zhang J., Anshul F., Malhotra D.K., Jaume J., Dworkin L.D., Gong R. (2021). Microdose Lithium Protects against Pancreatic Islet Destruction and Renal Impairment in Streptozotocin-Elicited Diabetes. Antioxidants.

[10] Gu Z.Y., Miao X.Y., Ma L.C., Gao J.J., Gong Y.P., Li C.L. (2021). Maintenance of cellular annexin A1 level is essential for PI3K/AKT/mTOR-mediated proliferation of pancreatic beta cells. J. Biol. Regul. Homeost. Agents.

[11] Moon J.H., Kim Y.G., Kim K., Osonoi S., Wang S., Saunders D.C., Wang J., Yang K., Kim H., Lee J. (2020). Serotonin Regulates Adult beta-Cell Mass by Stimulating Perinatal beta-Cell Proliferation. Diabetes.

[12] Sato T., Ishiwatari C., Kaneko Y.K., Ishikawa Y., Kimura Y., Watanabe N., Aoshima I., Matsuda Y., Nakayama T., Chiba R. (2021). Diacylglycerol kinase delta functions as a proliferation suppressor in pancreatic beta-cells. FASEB J..

[13] Kaise T., Fukui M., Sueda R., Piao W., Yamada M., Kobayashi T., Imayoshi I., Kageyama R. (2022). Functional rejuvenation of aged neural stem cells by Plagl2 and anti-Dyrk1a activity. Genes Dev..

[14] Yabut O., Domogauer J., D’Arcangelo G. (2010). Dyrk1A overexpression inhibits proliferation and induces premature neuronal differentiation of neural progenitor cells. J. Neurosci..

[15] Lindberg M.F., Meijer L. (2021). Dual-Specificity, Tyrosine Phosphorylation-Regulated Kinases (DYRKs) and cdc2-Like Kinases (CLKs) in Human Disease, an Overview. Int. J. Mol. Sci..

[16] Wang P., Alvarez-Perez J.C., Felsenfeld D.P., Liu H., Sivendran S., Bender A., Kumar A., Sanchez R., Scott D.K., Garcia-Ocana A. (2015). A high-throughput chemical screen reveals that harmine-mediated inhibition of DYRK1A increases human pancreatic beta cell replication. Nat. Med..

[17] Su Z., Ling X., Ji K., Huang H., Liu X., Yin C., Zhu H., Guo Y., Mo Y., Lu Y. (2020). (1)H NMR-based urinary metabonomic study of the antidiabetic effects of *Rubus suavissimus* S. Lee in STZ-induced T1DM rats. J. Chromatogr. B Anal. Technol. Biomed. Life Sci..

[18] Wu Y.L., Ding Y.P., Gao J., Tanaka Y., Zhang W. (2013). Risk factors and primary prevention trials for type 1 diabetes. Int. J. Biol. Sci..

[19] Rahier J., Guiot Y., Goebbels R.M., Sempoux C., Henquin J.C. (2008). Pancreatic beta-cell mass in European subjects with type 2 diabetes. Diabetes Obes. Metab..

[20] Eizirik D.L., Pasquali L., Cnop M. (2020). Pancreatic beta-cells in type 1 and type 2 diabetes mellitus: Different pathways to failure. Nat. Rev. Endocrinol..

[21] Younossi Z.M., Golabi P., de Avila L., Paik J.M., Srishord M., Fukui N., Qiu Y., Burns L., Afendy A., Nader F. (2019). The global epidemiology of NAFLD and NASH in patients with type 2 diabetes: A systematic review and meta-analysis. J. Hepatol..

[22] Hu H.Q., Qiao J.T., Liu F.Q., Wang J.B., Sha S., He Q., Cui C., Song J., Zang N., Wang L.S. (2020). The STING-IRF3 pathway is involved in lipotoxic injury of pancreatic beta cells in type 2 diabetes. Mol. Cell. Endocrinol..

[23] Chen K., Hua H., Zhu Z., Wu T., Jia Z., Liu Q. (2020). Artemisinin and dihydroartemisinin promote beta-cell apoptosis induced by palmitate via enhancing ER stress. Apoptosis.

[24] Leenders F., Groen N., de Graaf N., Engelse M.A., Rabelink T.J., de Koning E.J.P., Carlotti F. (2021). Oxidative Stress Leads to beta-Cell Dysfunction Through Loss of beta-Cell Identity. Front. Immunol..

[25] Fu A., Alvarez-Perez J.C., Avizonis D., Kin T., Ficarro S.B., Choi D.W., Karakose E., Badur M.G., Evans L., Rosselot C. (2020). Glucose-dependent partitioning of arginine to the urea cycle protects beta-cells from inflammation. Nat. Metab..

[26] Mukhuty A., Fouzder C., Mukherjee S., Malick C., Mukhopadhyay S., Bhattacharya S., Kundu R. (2017). Palmitate induced Fetuin-A secretion from pancreatic beta-cells adversely affects its function and elicits inflammation. Biochem. Biophys. Res. Commun..

[27] Rosselot C., Baumel-Alterzon S., Li Y., Brill G., Lambertini L., Katz L.S., Lu G., Garcia-Ocana A., Scott D.K. (2021). The many lives of Myc in the pancreatic beta-cell. J. Biol. Chem..

[28] Kaneto H., Sharma A., Suzuma K., Laybutt D.R., Xu G., Bonner-Weir S., Weir G.C. (2002). Induction of c-Myc expression suppresses insulin gene transcription by inhibiting NeuroD/BETA2-mediated transcriptional activation. J. Biol. Chem..

[29] Karslioglu E., Kleinberger J.W., Salim F.G., Cox A.E., Takane K.K., Scott D.K., Stewart A.F. (2011). cMyc is a principal upstream driver of beta-cell proliferation in rat insulinoma cell lines and is an effective mediator of human beta-cell replication. Mol. Endocrinol..

[30] Duchon A., Herault Y. (2016). DYRK1A, a Dosage-Sensitive Gene Involved in Neurodevelopmental Disorders, Is a Target for Drug Development in Down Syndrome. Front. Behav. Neurosci..

[31] Feki A., Hibaoui Y. (2018). DYRK1A Protein, A Promising Therapeutic Target to Improve Cognitive Deficits in Down Syndrome. Brain Sci..

[32] Dowjat K., Adayev T., Wojda U., Brzozowska K., Barczak A., Gabryelewicz T., Hwang Y.W. (2019). Abnormalities of DYRK1A-Cytoskeleton Complexes in the Blood Cells as Potential Biomarkers of Alzheimer’s Disease. J. Alzheimers Dis..

[33] Liu W., Liu X., Tian L., Gao Y., Liu W., Chen H., Jiang X., Xu Z., Ding H., Zhao Q. (2021). Design, synthesis and biological evaluation of harmine derivatives as potent GSK-3beta/DYRK1A dual inhibitors for the treatment of Alzheimer’s disease. Eur. J. Med. Chem..

[34] Cen L., Xiao Y., Wei L., Mo M., Chen X., Li S., Yang X., Huang Q., Qu S., Pei Z. (2016). Association of DYRK1A polymorphisms with sporadic Parkinson’s disease in Chinese Han population. Neurosci. Lett..

[35] Bai Z., Du Y., Cong L., Cheng Y. (2020). The USP22 promotes the growth of cancer cells through the DYRK1A in pancreatic ductal adenocarcinoma. Gene.

[36] Malinge S., Bliss-Moreau M., Kirsammer G., Diebold L., Chlon T., Gurbuxani S., Crispino J.D. (2012). Increased dosage of the chromosome 21 ortholog Dyrk1a promotes megakaryoblastic leukemia in a murine model of Down syndrome. J. Clin. Investig..

[37] Tarpley M., Oladapo H.O., Strepay D., Caligan T.B., Chdid L., Shehata H., Roques J.R., Thomas R., Laudeman C.P., Onyenwoke R.U. (2021). Identification of harmine and beta-carboline analogs from a high-throughput screen of an approved drug collection; profiling as differential inhibitors of DYRK1A and monoamine oxidase A and for in vitro and in vivo anti-cancer studies. Eur. J. Pharm. Sci..

[38] Booiman T., Loukachov V.V., van Dort K.A., van‘t Wout A.B., Kootstra N.A. (2015). DYRK1A Controls HIV-1 Replication at a Transcriptional Level in an NFAT Dependent Manner. PLoS ONE.

[39] Hutterer C., Milbradt J., Hamilton S., Zaja M., Leban J., Henry C., Vitt D., Steingruber M., Sonntag E., Zeittrager I. (2017). Inhibitors of dual-specificity tyrosine phosphorylation-regulated kinases (DYRK) exert a strong anti-herpesviral activity. Antivir. Res..

[40] Rozen E.J., Roewenstrunk J., Barallobre M.J., Di Vona C., Jung C., Figueiredo A.F., Luna J., Fillat C., Arbones M.L., Graupera M. (2018). DYRK1A Kinase Positively Regulates Angiogenic Responses in Endothelial Cells. Cell Rep..

[41] Ren D., Sun J., Mao L., Ye H., Polonsky K.S. (2014). BH3-only molecule Bim mediates beta-cell death in IRS2 deficiency. Diabetes.

[42] Catalano-Iniesta L., Iglesias-Osma M.C., Sanchez-Robledo V., Carretero-Hernandez M., Blanco E.J., Carretero J., Garcia-Barrado M.J. (2018). Variations in adrenal gland medulla and dopamine effects induced by the lack of Irs2. J. Physiol. Biochem..

[43] Mao N., Gao D., Hu W., Gadal S., Hieronymus H., Wang S., Lee Y.S., Sullivan P., Zhang Z., Choi D. (2020). Oncogenic ERG Represses PI3K Signaling through Downregulation of IRS2. Cancer Res..

[44] Lu M., Ma L., Shan P., Liu A., Yu X., Jiang W., Wang X., Zhao X., Ye X., Wang T. (2019). DYRK1A aggravates beta cell dysfunction and apoptosis by promoting the phosphorylation and degradation of IRS2. Exp. Gerontol..

[45] Lang L., Pettko-Szandtner A., Tuncay Elbasi H., Takatsuka H., Nomoto Y., Zaki A., Dorokhov S., de Jaeger G., Eeckhout D., Ito M. (2021). The DREAM complex represses growth in response to DNA damage in Arabidopsis. Life Sci. Alliance.

[46] Fajas L., Annicotte J.S., Miard S., Sarruf D., Watanabe M., Auwerx J. (2004). Impaired pancreatic growth, beta cell mass, and beta cell function in E2F1 (-/-)mice. J. Clin. Investig..

[47] Litovchick L., Florens L.A., Swanson S.K., Washburn M.P., DeCaprio J.A. (2011). DYRK1A protein kinase promotes quiescence and senescence through DREAM complex assembly. Genes Dev..

[48] Lee J.G., Kay E.P. (2011). PI 3-kinase/Rac1 and ERK1/2 regulate FGF-2-mediated cell proliferation through phosphorylation of p27 at Ser10 by KIS and at Thr187 by Cdc25A/Cdk2. Investig. Ophthalmol. Vis. Sci..

[49] Soppa U., Schumacher J., Florencio Ortiz V., Pasqualon T., Tejedor F.J., Becker W. (2014). The Down syndrome-related protein kinase DYRK1A phosphorylates p27(Kip1) and Cyclin D1 and induces cell cycle exit and neuronal differentiation. Cell Cycle.

[50] Abdolazimi Y., Zhao Z., Lee S., Xu H., Allegretti P., Horton T.M., Yeh B., Moeller H.P., Nichols R.J., McCutcheon D. (2018). CC-401 Promotes beta-Cell Replication via Pleiotropic Consequences of DYRK1A/B Inhibition. Endocrinology.

[51] Fatrai S., Elghazi L., Balcazar N., Cras-Meneur C., Krits I., Kiyokawa H., Bernal-Mizrachi E. (2006). Akt induces beta-cell proliferation by regulating cyclin D1, cyclin D2, and p21 levels and cyclin-dependent kinase-4 activity. Diabetes.

[52] Polager S., Ginsberg D. (2008). E2F—At the crossroads of life and death. Trends Cell Biol..

[53] Huang B., Zhu W., Zhao H., Zeng F., Wang E., Wang H., Chen J., Li M., Huang C., Ren L. (2020). Placenta-Derived Osteoprotegerin Is Required for Glucose Homeostasis in Gestational Diabetes Mellitus. Front. Cell Dev. Biol..

[54] Kondegowda N.G., Fenutria R., Pollack I.R., Orthofer M., Garcia-Ocana A., Penninger J.M., Vasavada R.C. (2015). Osteoprotegerin and Denosumab Stimulate Human Beta Cell Proliferation through Inhibition of the Receptor Activator of NF-kappaB Ligand Pathway. Cell Metab..

[55] Cebrian A., Garcia-Ocana A., Takane K.K., Sipula D., Stewart A.F., Vasavada R.C. (2002). Overexpression of parathyroid hormone-related protein inhibits pancreatic beta-cell death in vivo and in vitro. Diabetes.

[56] Guthalu Kondegowda N., Joshi-Gokhale S., Harb G., Williams K., Zhang X.Y., Takane K.K., Zhang P., Scott D.K., Stewart A.F., Garcia-Ocana A. (2010). Parathyroid hormone-related protein enhances human ss-cell proliferation and function with associated induction of cyclin-dependent kinase 2 and cyclin E expression. Diabetes.

[57] El Ouaamari A., Dirice E., Gedeon N., Hu J., Zhou J.Y., Shirakawa J., Hou L., Goodman J., Karampelias C., Qiang G. (2016). SerpinB1 Promotes Pancreatic beta Cell Proliferation. Cell Metab..

[58] Stephens S.B., Schisler J.C., Hohmeier H.E., An J., Sun A.Y., Pitt G.S., Newgard C.B. (2012). A VGF-derived peptide attenuates development of type 2 diabetes via enhancement of islet beta-cell survival and function. Cell Metab..

[59] Untereiner A., Xu J., Bhattacharjee A., Cabrera O., Hu C., Dai F.F., Wheeler M.B. (2020). gamma-aminobutyric acid stimulates beta-cell proliferation through the mTORC1/p70S6K pathway, an effect amplified by Ly49, a novel gamma-aminobutyric acid type A receptor positive allosteric modulator. Diabetes Obes. Metab..

[60] Zhang L., Li D., Yu S. (2020). Pharmacological effects of harmine and its derivatives: A review. Arch. Pharm. Res..

[61] Ogawa Y., Nonaka Y., Goto T., Ohnishi E., Hiramatsu T., Kii I., Yoshida M., Ikura T., Onogi H., Shibuya H. (2010). Development of a novel selective inhibitor of the Down syndrome-related kinase Dyrk1A. Nat. Commun..

[62] Shen W., Taylor B., Jin Q., Nguyen-Tran V., Meeusen S., Zhang Y.Q., Kamireddy A., Swafford A., Powers A.F., Walker J. (2015). Inhibition of DYRK1A and GSK3B induces human beta-cell proliferation. Nat. Commun..

[63] Dirice E., Walpita D., Vetere A., Meier B.C., Kahraman S., Hu J., Dancik V., Burns S.M., Gilbert T.J., Olson D.E. (2016). Inhibition of DYRK1A Stimulates Human beta-Cell Proliferation. Diabetes.

[64] Allegretti P.A., Horton T.M., Abdolazimi Y., Moeller H.P., Yeh B., Caffet M., Michel G., Smith M., Annes J.P. (2020). Generation of highly potent DYRK1A-dependent inducers of human beta-Cell replication via Multi-Dimensional compound optimization. Bioorg. Med. Chem..

[65] Kumar K., Wang P., Sanchez R., Swartz E.A., Stewart A.F., DeVita R.J. (2018). Development of Kinase-Selective, Harmine-Based DYRK1A Inhibitors that Induce Pancreatic Human beta-Cell Proliferation. J. Med. Chem..

[66] Kumar K., Wang P., Wilson J., Zlatanic V., Berrouet C., Khamrui S., Secor C., Swartz E.A., Lazarus M., Sanchez R. (2020). Synthesis and Biological Validation of a Harmine-Based, Central Nervous System (CNS)-Avoidant, Selective, Human beta-Cell Regenerative Dual-Specificity Tyrosine Phosphorylation-Regulated Kinase A (DYRK1A) Inhibitor. J. Med. Chem..

[67] Li X., Li S., Chen M., Wang J., Xie B., Sun Z. (2018). (-)-Epigallocatechin-3-gallate (EGCG) inhibits starch digestion and improves glucose homeostasis through direct or indirect activation of PXR/CAR-mediated phase II metabolism in diabetic mice. Food Funct..

[68] Mi Y., Liu X., Tian H., Liu H., Li J., Qi G., Liu X. (2018). EGCG stimulates the recruitment of brite adipocytes, suppresses adipogenesis and counteracts TNF-alpha-triggered insulin resistance in adipocytes. Food Funct..

[69] Zheng M., Zhang Q., Zhang C., Wu C., Yang K., Song Z., Wang Q., Li C., Zhou Y., Chen J. (2021). A natural DYRK1A inhibitor as a potential stimulator for beta-cell proliferation in diabetes. Clin. Transl. Med..

[70] Loaec N., Attanasio E., Villiers B., Durieu E., Tahtouh T., Cam M., Davis R.A., Alencar A., Roue M., Bourguet-Kondracki M.L. (2017). Marine-Derived 2-Aminoimidazolone Alkaloids. Leucettamine B-Related Polyandrocarpamines Inhibit Mammalian and Protozoan DYRK & CLK Kinases. Mar. Drugs.

[71] Choi M., Kim A.K., Ham Y., Lee J.Y., Kim D., Yang A., Jo M.J., Yoon E., Heo J.N., Han S.B. (2021). Aristolactam BIII, a naturally derived DYRK1A inhibitor, rescues Down syndrome-related phenotypes. Phytomedicine.

[72] Brial F., Alzaid F., Sonomura K., Kamatani Y., Meneyrol K., Le Lay A., Pean N., Hedjazi L., Sato T.A., Venteclef N. (2020). The Natural Metabolite 4-Cresol Improves Glucose Homeostasis and Enhances beta-Cell Function. Cell Rep..

[73] Zhao C., Wang D., Gao Z., Kan H., Qiu F., Chen L., Li H. (2020). Licocoumarone induces BxPC-3 pancreatic adenocarcinoma cell death by inhibiting DYRK1A. Chem. Biol. Interact..

[74] Liu X., Li M., Tan S., Wang C., Fan S., Huang C. (2017). Harmine is an inflammatory inhibitor through the suppression of NF-kappaB signaling. Biochem. Biophys. Res. Commun..

[75] Ding Y., He J., Huang J., Yu T., Shi X., Zhang T., Yan G., Chen S., Peng C. (2019). Harmine induces anticancer activity in breast cancer cells via targeting TAZ. Int. J. Oncol..

[76] Frost D., Meechoovet B., Wang T., Gately S., Giorgetti M., Shcherbakova I., Dunckley T. (2011). beta-carboline compounds, including harmine, inhibit DYRK1A and tau phosphorylation at multiple Alzheimer’s disease-related sites. PLoS ONE.

[77] He D., Wu H., Wei Y., Liu W., Huang F., Shi H., Zhang B., Wu X., Wang C. (2015). Effects of harmine, an acetylcholinesterase inhibitor, on spatial learning and memory of APP/PS1 transgenic mice and scopolamine-induced memory impairment mice. Eur. J. Pharmacol..

[78] Mazur-Kolecka B., Golabek A., Kida E., Rabe A., Hwang Y.W., Adayev T., Wegiel J., Flory M., Kaczmarski W., Marchi E. (2012). Effect of DYRK1A activity inhibition on development of neuronal progenitors isolated from Ts65Dn mice. J. Neurosci. Res..

[79] Wang P., Karakose E., Liu H., Swartz E., Ackeifi C., Zlatanic V., Wilson J., Gonzalez B.J., Bender A., Takane K.K. (2019). Combined Inhibition of DYRK1A, SMAD, and Trithorax Pathways Synergizes to Induce Robust Replication in Adult Human Beta Cells. Cell Metab..

[80] Ackeifi C., Wang P., Karakose E., Manning Fox J.E., Gonzalez B.J., Liu H., Wilson J., Swartz E., Berrouet C., Li Y. (2020). GLP-1 receptor agonists synergize with DYRK1A inhibitors to potentiate functional human beta cell regeneration. Sci. Transl. Med..

[81] Khan N., Mukhtar H. (2018). Tea Polyphenols in Promotion of Human Health. Nutrients.

[82] Ortsater H., Grankvist N., Wolfram S., Kuehn N., Sjoholm A. (2012). Diet supplementation with green tea extract epigallocatechin gallate prevents progression to glucose intolerance in db/db mice. Nutr. Metab..

[83] Pournourmohammadi S., Grimaldi M., Stridh M.H., Lavallard V., Waagepetersen H.S., Wollheim C.B., Maechler P. (2017). Epigallocatechin-3-gallate (EGCG) activates AMPK through the inhibition of glutamate dehydrogenase in muscle and pancreatic ss-cells: A potential beneficial effect in the pre-diabetic state?. Int. J. Biochem. Cell Biol..

[84] Bain J., McLauchlan H., Elliott M., Cohen P. (2003). The specificities of protein kinase inhibitors: An update. Biochem. J..

[85] Wang H., Yuan X., Huang H., Zhang B., Cao C., Zhao H.P. (2017). Chemical constituents from *Swertia mussotii* Franch. (Gentianaceae). Nat. Prod. Res..

[86] Ni Y., Liu M., Yu H., Chen Y., Liu Y., Chen S., Ruan J., Da A., Zhang Y., Wang T. (2019). Desmethylbellidifolin From *Gentianella acuta* Ameliorate TNBS-Induced Ulcerative Colitis through Antispasmodic Effect and Anti-Inflammation. Front. Pharmacol..

[87] Burgy G., Tahtouh T., Durieu E., Foll-Josselin B., Limanton E., Meijer L., Carreaux F., Bazureau J.P. (2013). Chemical synthesis and biological validation of immobilized protein kinase inhibitory Leucettines. Eur. J. Med. Chem..

[88] Hu H., Lee-Fong Y., Peng J., Hu B., Li J., Li Y., Huang H. (2021). Comparative Research of Chemical Profiling in Different Parts of *Fissistigma oldhamii* by Ultra-High-Performance Liquid Chromatography Coupled with Hybrid Quadrupole-Orbitrap Mass Spectrometry. Molecules.

[89] Choi Y.L., Kim J.K., Choi S.U., Min Y.K., Bae M.A., Kim B.T., Heo J.N. (2009). Synthesis of aristolactam analogues and evaluation of their antitumor activity. Bioorg. Med. Chem. Lett..

[90] Chia Y.C., Chang F.R., Teng C.M., Wu Y.C. (2000). Aristolactams and dioxoaporphines from *Fissistigma balansae* and *Fissistigma oldhamii*. J. Nat. Prod..

[91] Meijers B.K., Bammens B., de Moor B., Verbeke K., Vanrenterghem Y., Evenepoel P. (2008). Free p-cresol is associated with cardiovascular disease in hemodialysis patients. Kidney Int..

[92] Wu L., Fan Y., Fan C., Yu Y., Sun L., Jin Y., Zhang Y., Ye R.D. (2017). Licocoumarone isolated from *Glycyrrhiza uralensis* selectively alters LPS-induced inflammatory responses in RAW 264.7 macrophages. Eur. J. Pharmacol..

[93] Zhang Y., Xu Y., Zhang L., Chen Y., Wu T., Liu R., Sui W., Zhu Q., Zhang M. (2022). Licorice extract ameliorates hyperglycemia through reshaping gut microbiota structure and inhibiting TLR4/NF-kappaB signaling pathway in type 2 diabetic mice. Food Res. Int..

[94] Rathwa N., Patel R., Palit S.P., Parmar N., Rana S., Ansari M.I., Ramachandran A.V., Begum R. (2020). beta-cell replenishment: Possible curative approaches for diabetes mellitus. Nutr. Metab. Cardiovasc. Dis..

[95] Li Y., Xie X., Jie Z., Zhu L., Yang J.Y., Ko C.J., Gao T., Jain A., Jung S.Y., Baran N. (2021). DYRK1a mediates BAFF-induced noncanonical NF-kappaB activation to promote autoimmunity and B-cell leukemogenesis. Blood.

[96] Bhansali R.S., Rammohan M., Lee P., Laurent A.P., Wen Q., Suraneni P., Yip B.H., Tsai Y.C., Jenni S., Bornhauser B. (2021). DYRK1A regulates B cell acute lymphoblastic leukemia through phosphorylation of FOXO1 and STAT3. J. Clin. Investig..

[97] Liu A., Zhang B., Zhao W., Tu Y., Wang Q., Li J. (2021). MicroRNA-215-5p inhibits the proliferation of keratinocytes and alleviates psoriasis-like inflammation by negatively regulating DYRK1A and its downstream signalling pathways. Exp. Dermatol..

[98] Arbones M.L., Thomazeau A., Nakano-Kobayashi A., Hagiwara M., Delabar J.M. (2019). DYRK1A and cognition: A lifelong relationship. Pharmacol. Ther..

[99] Masaki S., Kii I., Sumida Y., Kato-Sumida T., Ogawa Y., Ito N., Nakamura M., Sonamoto R., Kataoka N., Hosoya T. (2015). Design and synthesis of a potent inhibitor of class 1 DYRK kinases as a suppressor of adipogenesis. Bioorg. Med. Chem..

[100] Saunders D.C., Brissova M., Phillips N., Shrestha S., Walker J.T., Aramandla R., Poffenberger G., Flaherty D.K., Weller K.P., Pelletier J. (2019). Ectonucleoside Triphosphate Diphosphohydrolase-3 Antibody Targets Adult Human Pancreatic beta Cells for In Vitro and In Vivo Analysis. Cell Metab..

[101] Liu Q., Liu N., Zang S., Liu H., Wang P., Ji C., Sun X. (2014). Tumor suppressor DYRK1A effects on proliferation and chemoresistance of AML cells by downregulating c-Myc. PLoS ONE.

